# Long-Term Outcome of Differentiated Thyroid Cancer Patients—Fifty Years of Croatian Thyroid Disease Referral Centre Experience

**DOI:** 10.3390/diagnostics12040866

**Published:** 2022-03-30

**Authors:** Tomislav Jukić, Ivan Blažeković, Maja Franceschi, Petra Petranović Ovčariček, Marija Bosak Butković, Nina Dabelić, Roko Granić, Marija Punda, Zdenko Sonicki, Davor Vagić, Ana Fröbe, Zvonko Kusić

**Affiliations:** 1 Department of Oncology and Nuclear Medicine, Sestre Milosrdnice University Hospital Center, 10000 Zagreb, Croatia; blazekovic90@gmail.com (I.B.); maja.franceschi@gmail.com (M.F.); p.petranovic@gmail.com (P.P.O.); marija.bosak@yahoo.com (M.B.B.); nina.dabelic@kbcsm.hr (N.D.); roko.granic@gmail.com (R.G.); marijapunda@hotmail.com (M.P.); ana.frobe@kbcsm.hr (A.F.); 2 School of Medicine, University of Zagreb, 10000 Zagreb, Croatia; zdenko.sonicki@snz.hr (Z.S.); davor.vagic@kbcsm.hr (D.V.); 3 Faculty of Medicine Osijek, Josip Juraj Strossmayer University of Osijek, 31000 Osijek, Croatia; 4 School of Public Health “Andrija Štampar”, 10000 Zagreb, Croatia; 5 Department of Otorinolaringology, Head and Neck Surgery, Sestre Milosrdnice University Hospital Center, 10000 Zagreb, Croatia; 6 School of Dental Medicine, University of Zagreb, 10000 Zagreb, Croatia; 7 Croatian Academy of Science and Arts, 10000 Zagreb, Croatia; zaklada@hazu.hr

**Keywords:** differentiated thyroid cancer, papillary, follicular, survival rates, prognostic features, recurrence, follow-up

## Abstract

Background: Indolent nature but a high incidence of differentiated thyroid cancer (DTC) remains a challenge for optimizing patient care. Therefore, prognostic factors present valuable information for determining an adequate clinical approach. Methods: This study assessed prognostic features of 1167 papillary (PTC) and 215 follicular (FTC) thyroid cancer patients that had undergone surgery between 1962 and 2012, and were followed-up up to 50 years in a single institution, till April 2020. Age, gender, tumor size, presence of local and distant metastases at presentation, extrathyroidal extension, disease recurrence, and cancer-specific survival were evaluated. Results: In multivariate analysis, factors affecting the worse outcome were age (*p* = 0.005), tumor size (*p* = 0.006), and distant metastases (*p* = 0.001) in PTC, while extrathyroidal extension (*p* < 0.001), neck recurrence (*p* = 0.002), and distant metastases (*p* < 0.001) in FTC patients. Loco-regional recurrence rate was 6% for PTC and 4.7% for FTC patients, while distant metastases were detected in 4.2% PTC and 14.4% of FTC patients. The 10-year cancer-specific survival rates for PTC and FTC were 98.6% and 89.8%, respectively (*p* < 0.001). Conclusions: Negative prognostic factors, besides distant metastases, were older age and greater tumor size in PTC, and extrathyroidal extension and neck recurrence in FTC patients. The recurrence and mortality rates were very low.

## 1. Introduction

Thyroid cancer is the most common endocrine malignancy. The worldwide challenge of thyroid cancer (TC) is manifested as approximately 1% of all new malignant neoplasms in men and 3.5% in the women population [1]. During the last few decades, an increase in the incidence of TC was recorded worldwide, in both men and women, while mortality rates in most countries remained stable or even declined [2]. According to Globocan Cancer Observatory data, Croatia is rated as the fourth country in Europe and the eleventh country in the world regarding the incidence of TC [1]. Thyroid cancer in Croatia is the most common cancer site in a female population aged 10–29 years and overall, the fifth most common cancer in females with four times greater incidence rate than males. The incidence rate is also following the trend of a constant rise in the total population [3]. To that extent, an increase in TC survival in Europe expresses a strong correlation between incidence and survival, both increasing over time, but without correlation between incidence and mortality [4]. The vast majority of the increase in TC incidence worldwide is due to the increase in PTC, mainly due to improved diagnostics [5]. More precisely, in 2009 the revised ATA guidelines suggested that even 5 to 10 mm nodules with suspicious US features (hypoechogenicity, microcalcifications, infiltrative margins, increased vascularity, and nodules that have a taller-than-wide shape) should undergo fine-needle aspiration biopsy (FNAB) [6]. In line with that are the results of a study that presented a series of 475 thyroid FNAB of lesions ≤ 1 cm, and documented that 57.2% of suspected subcentimeter lesions were malignant [7]. PTC is the most common histologic form accounting for almost 80–85% of all TC cases, and FTC accounts for 10% of TCs [8]. PTCs and FTCs as differentiated thyroid cancers (DTC) are derived from follicular cells. The treatment strategy is similar for both cancer types, while the outcome is slightly better in PTC. However, clinical presentation differs. Up to 60% of PTC patients may have cervical nodal metastases and up to 20% multiple tumor sites within the thyroid. PTC invades lymphatic and blood vessels. Distant metastases of PTC are rare, accounting for less than 10% of PTC patients, most commonly in lungs and bones. FTC is characterized by a hematogenous spread with less common regional cervical nodal and more common distant metastases than PTC. Up to 25% FTCs patients may have distant metastases, most commonly in bones and lungs. Disease-specific 10-year mortality of DTC patients is less than 5%. However, recurrence rates are as high as 30% [8].

A unique property of TC is age-dependent staging of disease according to various staging systems. Studies have demonstrated age as a predictive factor independently associated with a better prognosis for younger patients [9,10]. The previous edition of tumor-node-metastasis (TNM) classification adopted a 45 year age limit [11]. Mayo Clinic MACIS scoring system (Metastases, Age, Completeness of resection, Invasion, Size) adopted a 40 year age limit [12], and Cancer Institute Hospital in Tokyo scoring system (CIH) classification determined a limit at 50 years [13]. Intraoperative staging system (iStage) proposed age cut-off at 55 years [14], as well as the recent Eight Edition of TNM AJCC/UICC scoring system [15]. Age, metastases, extent, size (AMES) prognostic scoring system provided a gender-specific age of 40 years for men and 50 years for women [16]. In clinical practice, TNM AJCC/UICC and MACIS systems demonstrated superiority predicting DTC patients outcomes [17]. Nowadays, TNM AJCC/UICC prognostic system is widely accepted in everyday practice.

Population studies indicate a greater incidence of DTC in the female population [1,3]. However, gender as a prognostic factor has not been confirmed. Recent studies suggest that gender is not a straightforward outcome predictor. Notably, when using gender as a predicting factor, age is necessary information [18]. PTC and FTC have a similar outcome in male patients. For PTC patients younger than 55 years, women had significantly better survival outcomes compared with men. However, there was no difference in survival outcomes between female and male patients older than 55 years. Outcomes of FTC patients are not affected by gender [18]. The size of primary tumor is profoundly discussed as a prognostic factor for DTC, and furthermore, as an important factor concerning indications and extent of surgical treatment. Previous differences in opinion, regarding the impact of primary tumor size on prognosis and surgical treatment, between Eastern and Western countries, are now getting less distinctive. Total or near-total thyroidectomy, radioiodine therapy (RAI), and L-thyroxine (L-T4) suppressive therapy have been standard treatment strategies for decades in most of the patients with DTC [8]. Croatian Thyroid Society Guidelines for the management of DTC patients [19] adopted European Thyroid Association (ETA) guidelines published in 2006 [20]. Following the European guidelines, total thyroidectomy and RAI were recommended for all patients with DTC except for patients with papillary microcarcinoma (tumor size ≤ 1 cm, without regional and distant metastases, and extrathyroidal extension). Japanese guidelines recommend total thyroidectomy only for high-risk patients with a tumor size larger than 5 cm in diameter. The consensus is made for other patients that total thyroidectomy is encouraged for tumors larger than 4 cm or those with regional lymph node metastases [21]. Previous American Thyroid Association (ATA) guidelines suggested total or near-total thyroidectomy for almost all patients with PTC ≥ 1 cm [6]. The 2015 ATA guidelines propose that patients with DTC > 1 cm and < 4 cm, without clinical evidence of lymph node metastases and extrathyroidal extension can be treated surgically either by total/near-total thyroidectomy or lobectomy alone. A less aggressive treatment approach in DTC patients regarding I-131 therapy as well as L-T4 suppressive therapy is also recommended [17]. However, total thyroidectomy enables RAI, subsequent risk stratification with iodine-131 whole-body scintigraphy, and facilitates follow-up [22]. According to risk for recurrence, DTC patients are classified into low, intermediate, and high-risk groups. Iodine-131 therapy is recommended for high-risk patients, may be considered in intermediate-risk patients, and is not routinely recommended in low-risk patients with a selective approach in this group [17]. However, a more personalized approach is recommended by European authors taking into consideration disease features or patient preferences [23]. There is no doubt that increasing tumor size is associated with a less favorable prognosis and higher mortality rates. Currently available guidelines and preferable staging TNM system point out that tumor size > 4 cm is an indicator of less favorable prognosis as well as extensive extrathyroidal extension and age [24]. Over the years, various studies provided a different cut-off point for tumor size as a prognostic factor. Ito et al. [25] concluded that tumor size > 2 cm is an independent factor for disease recurrence while Lang et al. [26] determined that the optimal cut-off point for tumor size is 3.5 cm.

Most mentioned and currently used scoring systems and guidelines do not take into account a specific difference between PTC and FTC. However, due to differences in clinicopathological features, different staging systems for PTCs and FTCs may be considered in the future [26].

The aim of the study was to evaluate prognostic features of PTC and FTC. Additionally, we aimed to present different clinicopathological patterns of PTC and FTC patients at the first onset of the disease, tumor recurrence rates, and disease-specific survival. Risk factors for local recurrence in PTC and FTC patients were assessed. Presented data encompass 50 years of follow-up of DTC patients in the Croatian Thyroid Diseases Referral Centre.

## 2. Materials and Methods

This retrospective study comprised 1382 patients with DTC. All patients were primarily treated from 1962 to 2012 and followed until April 2020 in Sestre milosrdnice University Hospital Centre, Zagreb, Referral Centre for Thyroid Diseases of the Ministry of Health. There were 1167 patients (84.4%) with a histological diagnosis of PTC and 215 patients (15.6%) with FTC. All histological types of PTC were included in the study. Hürthle cell tumors were excluded as a new category according to the 4th edition of the World Health Organization Histologic Classification of Thyroid Tumours. Demographic, clinical, pathohistological, treatment, and follow–up data of DTC patients were regularly registered in the referral center computer database. Personal records of inpatient and outpatient visits were used as a data source. The mean follow-up period of PTC patients was 12.0 years (0.2–48.1) and of FTC patients 11.0 years (0.4–45.5). We followed 906 (78%) PTC and 146 (68%) FTC patients for more than 5 years, 691 (59%) PTC and 97 (45%) FTC patients for more than 10 years, 145 (12%) PTC and 39 (18%) FTC pts for more than 20 years. In total, only 6 FTCs and 19 PTCs had follow-ups less than one year.

Demographic and clinical data of DTC patients are presented in Table 1. Both groups had similar sex and age characteristics. For PTC patients, the female vs. male ratio was 4:1 and the median age was 47 years (range 6–83 years). For FTC patients, the female vs. male ratio was 2.8:1 with a median age of 48 years (range 13–81 years). In most patients, differentiated thyroid cancer was suspected according to FNAB finding, or detected as an incidental pathohistological finding after surgery due to multinodular goiter or hyperthyroidism. In a few patients, PTC was detected after the removal of an enlarged neck lymph node, and subsequent histopathology revealed PTC neck metastasis, and total thyroidectomy with neck dissection was subsequently performed. In a few FTC patients, biopsy of bone metastasis revealed metastatic FTC, and subsequently, total thyroidectomy was performed. Almost all patients were surgically treated with total or near-total thyroidectomy, except 3 PTC patients treated with lobectomy alone and 2 PTC patients with thyroglossal duct PTC treated only with thyroglossal duct excision, and 1 FTC patient treated with lobectomy alone. Prior to surgery, neck ultrasound (US) and FNAB of suspicious cervical lymph nodes, and after 2005, FNAB combined with thyroglobulin (Tg) measurement in the aspirate samples of suspicious lymph nodes were performed. Computed tomography (CT) or magnetic resonance imaging (MRI) of the neck and thoracic region were performed preoperatively in patients with advanced tumors to evaluate the stage of disease and guide the extent of surgical treatment. After a minimum of 4 weeks after surgery, without L-T4 suppressive therapy, clinical examination, serum Tg and thyroglobulin antibodies (TgAb) measurement [25], neck US and, in most of the patients diagnostic I-131 whole-body scans (DxWBS) were obtained. According to initial risk stratification based on age and histopathology finding (TNM stage), serum Tg, TgAb, neck US, and DxWBS, while concerning available ETA [20] and Croatian Thyroid Society guidelines [19] for the management of patients with DTC, the treatment and follow-up decision was established regarding the patient’s management by a team of nuclear medicine specialists and oncologist. The patients were referred to radioiodine ablation/adjuvant therapy with activities ranging from 1110 to 5550 MBq (30–150 mCi) [27,28] or radioiodine therapy of known disease with I-131 activities ranging from 3700 to 9250 MBq (100–250 mCi). Radioiodine therapy in patients with distant metastases was administered periodically in intervals from 4 to 8 months.

Before 2005, radioiodine ablation was applied in all patients, with the exception of patients with lobectomy or excision of thyroglossal duct PTC and intact thyroid gland. After 2005, radioiodine (RAI) ablation was considered in patients with primary tumor size ≥ 1 cm, unfavorable histological type of DTC, incomplete resection, presence of extrathyroidal extension, positive cervical lymph nodes, or distant metastases, taking into account results of diagnostic procedures and Tg and TgAb. Overall, 1084/1167 (92.9%) PTC patients and 210/215 (97.7%) FTC patients underwent I-131 ablation/therapy. Radioiodine diagnostic whole-body scan was performed in most patients and changed management in 20% of patients regarding I-131 ablation activity and/or decision to treat.

A post-therapeutic whole-body scan (RxWBS) was performed and the initial disease stage was assessed. All patients were stratified according to the recent 8th ed. of TNM AJCC/UICC scoring system [15]. In patients with locally advanced TC (T4 tumors) and/or significant extranodal extension external beam radiation therapy was applied, as well as in patients with DTC bone metastases. After confirmation of thyroid remnant ablation with Tg, TgAb, and DxWBS, patients were followed-up at 6–12-month intervals with serum TSH, Tg and TgAb measurement, and neck US [29]. Patients with distant metastases were followed-up with DxWBS, RxWBS, and various radiological and nuclear medicine procedures: 99m-Tc MDP bone scintigraphy, US, chest X-ray, CT, MR, ^18^F-FDG PET/CT. If there was US suspicion of loco-regional recurrence, FNAB combined with Tg measurement in the aspirate samples was performed [30]. If DTC recurrence was suspected due to serum Tg rise, I-131 therapy was applied and RxWBS, to assess disease stage. If RxWBS was negative, CT was performed and after 2008, ^18^F-FDG PET/CT in all patients with elevated Tg level and negative RxWBS. In radioiodine refractory DTC patients chemotherapy, surgery, and redifferentiation therapies were applied. Only the first tumor recurrence was included in the analysis.

### Statistical Methods

Potential clinical and histopathological risk factors associated with survival (age, tumor size, gender, TNM disease stage, extrathyroidal extension, presence of distant and local metastases as well as local recurrence and new sites of distant metastases) were evaluated by univariate analysis according to Kaplan–Meier estimate. Survival time and cause-specific survival rates were presented as standard Kaplan–Meier survival curves with a subgroup correlation using the log-rank test. Risk factors for local recurrence were evaluated with univariate and multivariate analysis. Continuous variables (age and tumor size) were transformed into dichotomous variables. A multivariate Cox proportional hazard regression model was used to identify independent prognostic factors and their hazard effect.

To compare risk factors between PTCs and FTCs Pearson’s Chi-Square and Fishers exact tests were used for dichotomous variables (outcome, presence of distant metastases; recurrence, gender) and the Mann–Whitney U-test for continuous variables (age, tumor size). For continuous variables, the Kolmogorov–Smirnov test was used for assessing if data are normally distributed. In all analyses, *p*-values less than 0.05 were used as the cut-off for significance.

## 3. Results

Demographic and clinical data of 1382 patients treated and followed-up for differentiated thyroid cancer and differences between PTC and FTC patients are presented in Table 1.

At initial presentation, regional cervical nodal metastases were recorded in 254 (26.4%) PTC and 18 (14,8%) FTC patients (*p* = 0.005), and distant metastases were recorded in 41 (3.5%) PTC and 29 (13.5%) of FTC patients (*p* < 0.001). Multifocal tumors were recorded in 42.6% PTC patients and 5.4% FTC patients (*p* < 0.001) and extrathyroidal extension in 17.5% PTC patients and 32.1% FTC patients (*p* = 0.032).

Median tumor size was 12 (1–100) mm in PTC, and 32 (7–90) mm in FTC patients (*p* < 0.001). According to AJCC 8th ed., 1033 (88.6%) PTC patients and 168 (78.1%) FTC patients were classified as stage I. However, stage IVb was recorded in 17 (1.5%) PTC patients and 23 (10.7%) FTC patients (*p* < 0.001). There was no difference in gender and age between the PTC and FTC patients (Table 1).

**Figure 1:**
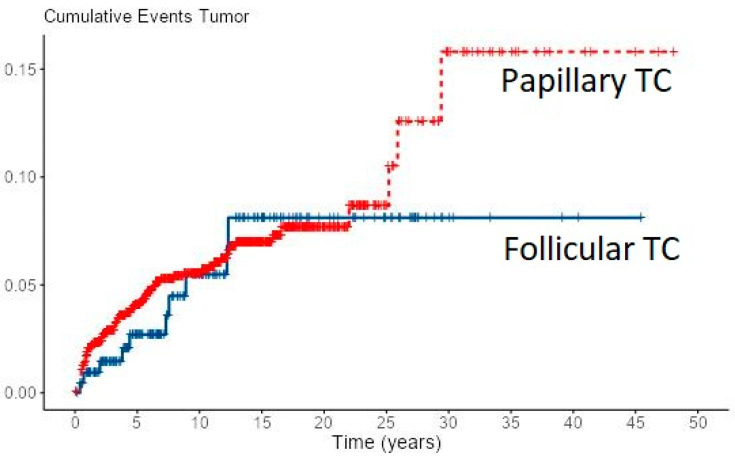
Cumulative events for local recurrence of PTC and FTC.

**Figure 2:**
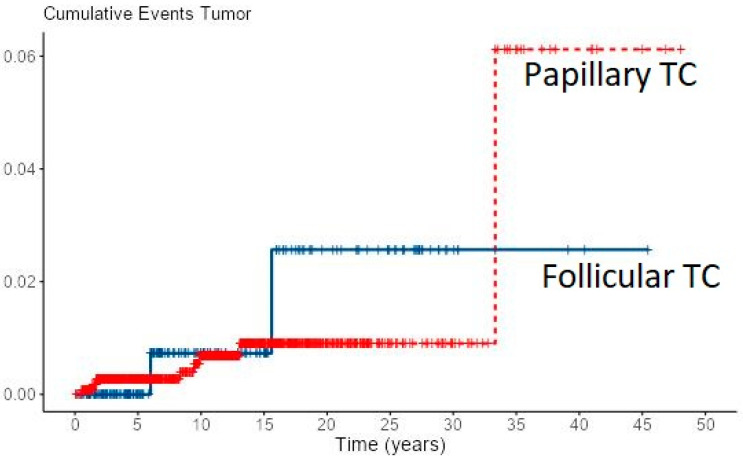
Cumulative events for new distant metastases of PTC and FTC.

Loco-regional recurrence was confirmed in 70 (6%) PTC and 10 (4.7%) FTC patients. During follow-up, new distant metastases were recorded in 8 PTC and 2 FTC patients, all in the lungs, accounting for overall 49 (4.2%) PTC patients and 31 (14.4%) of FTC patients with distant metastases. Most of the local recurrences as well as new distant metastases developed in the first 5–10 years of follow-up for both cancer types, especially in FTC patients (Figure 1 and Figure 2). However, in some PTC patients, local recurrence developed after 15 to 30 years of follow-up as well as distant metastases in a few PTC patients. In all eight PTC patients and two FTC patients, lung metastases developed during follow-up. In patients with distant metastases, the median tumor size was 25 mm in PTC patients vs. 64 mm in FTC patients. The female to male ratio in patients with distant metastases was 1.2:1 in PTC patients vs. 1.8:1 in FTC patients.

**Figure 3:**
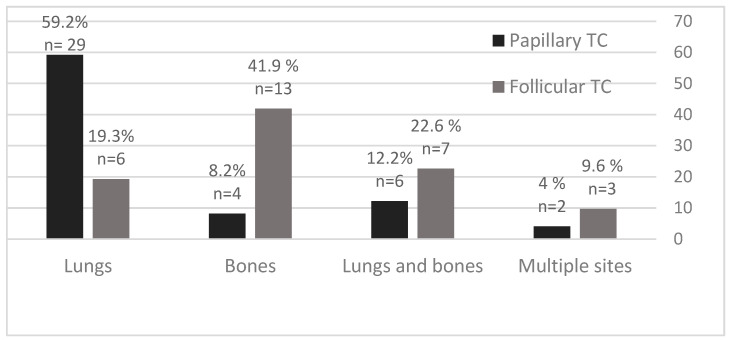
Distant metastases of PTC and FTC in percentage (%) and number according to the site of metastasis.

The most common sites of DTC distant metastases were lungs and bones, with specified differences between PTC and FTC patients (Figure 3). In PTC patients, the predominant site of distant metastases was the lungs in 29/49 (59.2%) patients, followed by lungs and bones in 6/49 (12.2%) patients, and bones in 4/49 (8.2%) patients. In FTC patients, the predominant site of distant metastases was in bones in 13/31 (41.9%) patients, followed by bones and lungs in 7/31 (22.6%) patients, and lungs in 6/31 (19.3%) patients.

During follow-up, 14 PTC and 20 FTC patients had a fatal outcome. In PTC patients with fatal outcome, the median tumor size and F/M ratio were 45 mm and 0.6:1, and in FTC patients 60 mm and 1.2:1. In eight PTC patients, a fatal outcome was due to multiple distant metastases while six PTC patients died due to inoperable advanced loco-regional disease. The fatal outcome in 17 FTC patients was due to extensive, most commonly bone metastases, while 3 patients died from the advanced loco-regional disease. Progression of distant metastases was recorded in 11 patients (3 PTC and 8 FTC patients, respectively).

**Figure 4:**
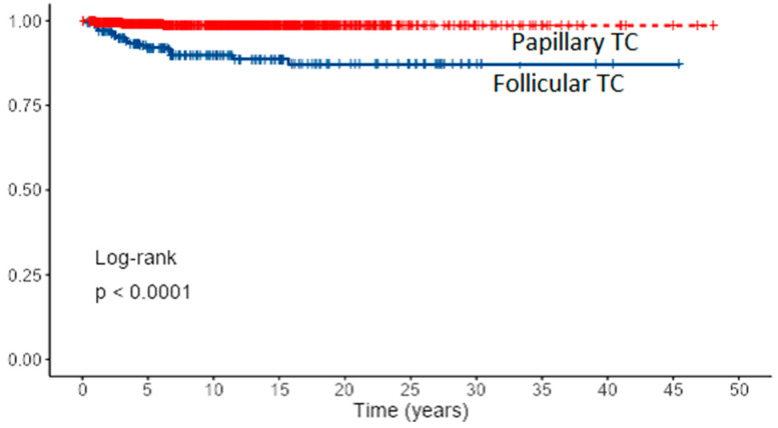
Survival curves for PTC and FTC patients based on Kaplan–Meier estimates.

**Figure 5:**
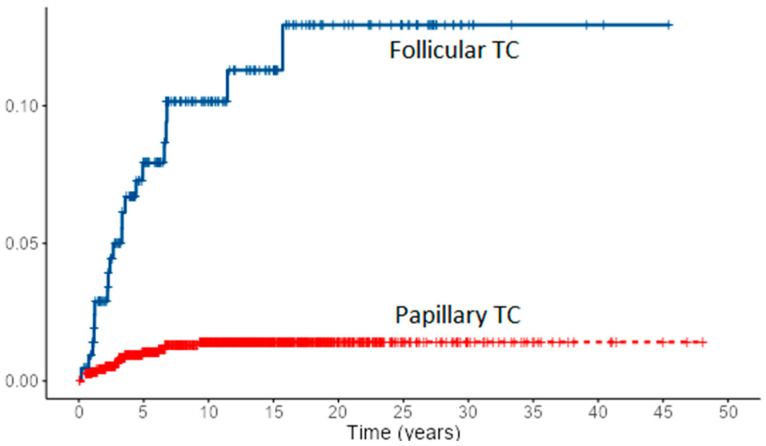
Cumulative death events (hazard) for PTC and FTC patients.

Overall, the 5- and 10-year cancer-specific survival rates were 6.1% and 98.6% for PTC (80% with distant metastases) and 92.7 and 89.8% for FTC (32% with distant metastases) (*p* < 0.001). Survival curves for PTC and FTC patients based on Kaplan–Meier estimates are presented in Figure 4 and cumulative death events in Figure 5.

**Figure 6:**
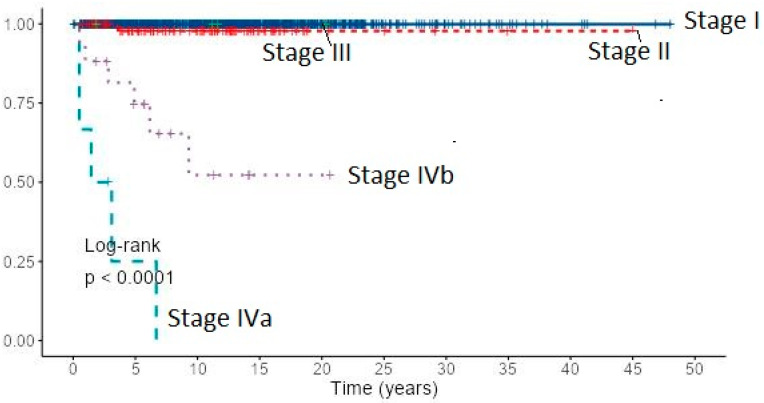
Cancer-specific survival of 1167 patients with PTC by the AJCC/UICC TNM staging system (8th edition) based on Kaplan–Meier estimates.

Cancer-specific 10-year survival for PTC and FTC patients by the AJCC/UICC TNM staging system (8th edition) was almost 100% in stage I for both cancer types, stage II: 97.8% for PTC and 94.4% for FTC, stage IVb: 74.0% and 52.3% for PTC and 38.0% and 15.2% for FTC, respectively (Table 2). Due to the small number of stage III patients, they were excluded from analysis. Cancer-specific survival of 1167 patients with PTC by the AJCC/UICC TNM staging system (8th edition) based on Kaplan–Meier estimates is presented in Figure 6.

Univariate analysis of potential clinicopathological factors influencing survival demonstrated all eight prognostic factors associated both with PTC and FTC: age, gender, tumor size, lymph node metastases, extrathyroidal extension, distant metastases, TNM stage IV, and local recurrence (Table 3). New sites of distant metastases (*p* < 0.001 and TNM stage II (*p* = 0.014) as predictors of outcome were associated only with PTC. Interestingly, TNM stage II showed no significance as a prognostic factor in FTC patients (*p* = 0.630). Lymph node metastases (*p* = 0.012) and TNM stage II (*p* = 0.014) were less reliable predictors of outcome in PTC, while gender was a less reliable predictor in FTC patients (*p* = 0.044). Age was found to be a significant prognostic factor for both cancer types (*p* < 0.001). Furthermore, age “cut-off” point as prognostic “cut-off” for both cancer types was estimated. The “cut-off” value for PTC was 60 years and 65 years for FTC. Further analysis showed a statistically significant greater tumor size in FTC patients (Mann–Whitney U test, and Fischer’s Exact test 1-sided, *p* < 0.001) and significantly more presentations with distant metastasis (*p < 0.001).* Statistical analysis of “cut-off” point for tumor size as prognostic factor revealed a value of 33 mm for PTC and 55 mm for FTC patients. Multivariate analysis revealed age (*p* = 0.005), tumor size (*p* = 0.006), distant metastases (*p* = 0.001), and new sites of distant metastases (*p* = 0.002) as independent prognostic factors in PTC patients. In FTC patients, multivariate analysis revealed extrathyroidal extension (*p* < 0.001), neck recurrence (*p* = 0.002), and distant metastases (*p* < 0.001) as independent prognostic factors affecting survival. However, a large 95% confidence interval was found for distant metastases in FTC patients.

**Figure 7:**
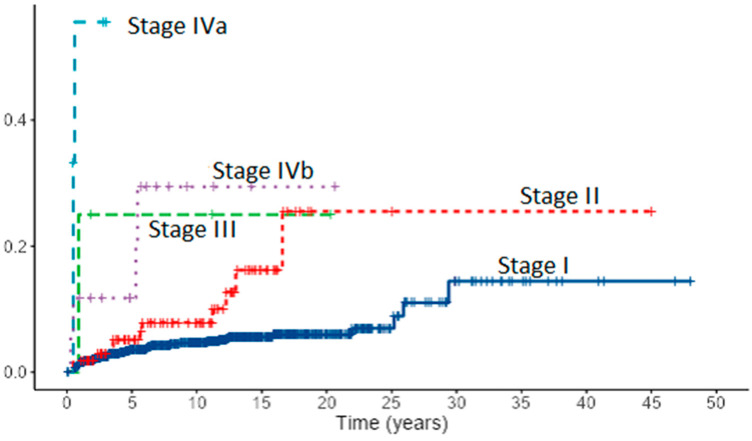
Cumulative events for local recurrence in 1167 patients with PTC by the AJCC/UICC TNM staging system (8th edition).

Assessment of risk factors for local recurrence revealed in PTC patients both with univariate and multivariate analysis lymph node metastases (*p* < 0.001), extrathyroidal extension (*p* < 0.001, *p* = 0.030), and stage IV (*p* < 0.001). Tumor size and age “cut-off” that increases the risk for recurrence in PTC patients was estimated to be 13 mm and 60 years. In FTC patients, lymph node metastases (*p* < 0.001), extrathyroidal extension (*p* < 0.001), distant metastases (*p* = 0.029), TNM stage (*p* < 0.001), age (*p* = 0.003) with “cut-off” point of 63 years and tumor size (*p* = 0.013) with “cut-off” point of *35 mm* appeared as risk factors for recurrence. However, in multivariate analysis, only lymph node metastases (*p* = 0.031) in FTC patients emerged as an independent risk factor for recurrence. Cumulative events for local recurrence in 1167 patients with PTC by the AJCC/UICC TNM staging system are presented in Figure 7.

## 4. Discussion

Differentiated thyroid cancers are malignant tumors with a very low mortality rate as demonstrated in this study, especially PTCs. Cancer-specific 5- and 10-year survival rates in our study were 99.1% and 98.6% for PTCs and 92.7 and 89.8% for FTCs. Most of the patients were treated with total or near-total thyroidectomy and radioiodine therapy as a standard of care. Patients with T4 tumors were additionally treated with external beam radiotherapy as reported in the literature [31]. Tsang et al. reported 10-year cancer-specific survival of 382 DTC patients with a median follow-up of 10.8 years of 93% for PTC patients and 69% for FTC patients [32]. Similar to our study, Cushing et al. reported overall and disease-specific 10-year survival of 333 DTC patients with a median follow-up of 10.4 years of 97.5% and 98.5%, respectively [33]. In our study, PTC patients with distant metastases had 80% cancer-specific 10-year survival. The most common sites of distant metastases in DTC patients were lungs and bones as reported in the literature [34,35]. The difference between PTC and FTC patients in the overall survival emerged due to more advanced tumor stages in FTC patients at presentation, and consequently, significantly worse disease-specific survival as previously reported by Mazzaferri et al. [36]. The worst outcome was reported in the AJCC/UICC TNM stage IVa group, due to large locally invasive recurrent tumors infiltrating adjacent neck structures, probably with dedifferentiation. However, the number of patients in this group was too small to draw relevant conclusions. There was no statistically significant difference in the survival of PTC and FTC patients with AJCC/UICC TNM stages I and II. Interestingly, a small number of patients with stage III was reported, probably due to the latest AJCC/UICC 8th TNM classification with an age cut-off of 55 years. Univariate analysis revealed all eight prognostic factors in DTC patients affecting survival: age, gender, tumor size, lymph node metastases, extrathyroidal extension, distant metastases, TNM stage, and local recurrence. New sites of distant metastases were prognostic factors only in PTC patients. It may be explained with a small number of events in the FTC patients group.

Lungs were the common site of distant recurrence. Multivariate analysis revealed age, tumor size, distant metastases, and new sites of distant metastases as independent prognostic factors in PTC patients. However, in FTC patients gross extrathyroidal extension, neck recurrence, and distant metastases emerged as independent prognostic factors. Age “cut–off “of 60 and older in PTC patients and 65 and older in FTC patients were associated with a higher risk of recurrence. The same age “cut-off” (>60), as a predictor of survival for DTC patients, was reported by Cushing et al. [33]. The percentage of local recurrence was 6.1% in PTC patients and 4.7% in FTC patients. Most of the recurrences appeared in the first 5–10 years of follow-up as previously reported by Mazzaferri et al. [36]. Risk factors for recurrence in PTC patients were lymph node metastases, capsular invasion, and AJCC/UICC TNM stage IV. In FTC patients, only lymph node metastases appeared as a risk factor for recurrence in multivariate analysis.

The strength of this study is in the long-term follow-up of a large cohort of DTC patients but with limiting factor that all patients were treated at one institution. We must take into consideration the long-time span of the study with differences in histopathology reporting, classifications, and diagnostics. However, the treatment strategy did not change significantly during the observed time period. Many of the FTC patients in the 1960s, 1970s, and 1980s had very large tumors invading neck structures and/or extensive bone metastases and therefore a high mortality rate as reported in the study. Diagnostic whole-body iodine-131 scintigraphy was used in postoperative risk assessment, determination of therapeutic I-131 activity, as well as early and in certain cases late follow-up. However, only in the last decade, we had a possibility in addition to planar imaging, to perform single-photon emission computed tomography/computed tomography which increases the sensitivity and specificity of the study.

Differentiated thyroid cancer patients have a generally favorable outcome, especially when discovered in earlier stages. Risk factors are well-known, as reported and confirmed in this study. The evaluation of the role of iodine-131 in the treatment of DTCs, especially intermediate-risk DTCs is a demanding task hard to resolve without long-term prospective studies. Our study was straightforward, because most of the patients received RIA for ablation, adjuvant therapy, or therapy of known disease, and evaluation of different treatment approaches cannot be evaluated. A systematic review of the literature supports postoperative use of RIA in DTC patients with tumors larger than 1 cm in diameter [37,38]. Our institution adopted the same approach after the year 2005. However, a less aggressive, selective approach regarding radioiodine ablation in low-risk patients with tumors 1–4 cm, confined to the thyroid, can be applied in certain cases, due to favorable outcome in this risk group. The question of follow-up strategy in DTC patients is another quest. An aggressive follow-up approach in more advanced tumor stages is preferable, while in DTC patients with early stages yearly follow-up strategy can be suggested. However, the duration of follow-up is hard to define. Late recurrences after 20–30 years are reported in this study, especially in stage I. Therefore, life-long follow-up of DTC patients with TSH, Tg, TgAb, and neck US is routinely advocated in our institution.

## Figures and Tables

**Table 1:** Demographic and clinical data of 1382 patients treated for differentiated thyroid cancer with analysis of differences between PTC and FTC.

	PTC	FTC
**N**	1167	215
Sex, F/M *n* (%)	934 (80%)/233 (20%)	158 (73%)/57 (27%)
Age at diagnosis, median (range) years	47 (6–83)	48 (13–81)
Tumor size, median (range) mm	12 (1–100) ***	32 (7–90) ***
Total thyroidectomy	1162	214
Lobectomy	3	1
Thyroglossal duct excision	2	0
Iodine-131 therapy *n* (%)	1084 (92.9%)	210 (97.7%)
Cervical nodal metastases et presentation	254/962 (26.4%) **	18/122 (14,8) **
Multifocality	256/601 (42.6%) ***	11/204 (5.4%) ***
Capsular invasion	125/714 (17.5%) *	18/56 (32.1%) *
Distant metastases at presentation	41 (3.5%) ***	29 (13.5%) ***
AJCC 8th edition		
I	1033 (88.6%)	168 (78.1%)
II	106 (9.1%)	21(9.8%)
III	4 (0.3%)	2 (0.9%)
IVa	6 (0.5%)	1 (0.5%)
IVb	17 (1.5%) ***	23 (10.7%) ***
Follow-up (median, range) years	12 (0.2–48)	11 (0.4–46)
Recurrence—neck Recurrence—distant	71 (6.1%) 8 (0.7%)	10 (4.7%) 2 (0.9%)
Outcome		
Died of disease	14 (1.2%) ***	20 (9.3%) ***
Persistent disease—local	33 (2.8%)	7 (3.3%)
Persistent disease—distant	16 (1.4%)	7 (3.3%)
Disease free	1104 (94.6%)	181 (84.1%)

Legend: * *p* < 0.05; ** *p* < 0.01; *** *p* < 0.001; F, female; M, male; PTC, papillary thyroid cancer; FTC, follicular thyroid cancer; AJCC, American Joint Committee on Cancer.

**Table 2:** Cancer-specific 5-year and 10-year survival (%) for PTC and FTC according to AJCC/UICC TNM staging system (8th edition).

TNM Stage	PTC		FTC	
	5-Year (%)	10-Year (%)	5-Year (%)	10-Year (%)
I	99.9	99.9	100	100
II	97.8	97.8	94.4	94.4
IVa	25.0	0	0	0
IVb	74.0	52.3	38.0	15.2

**Table 3:** Factors associated with survival in PTC and FTC patients by univariate analysis (Kaplan–Meier survival method, Log rank test).

Factors Associated with Survival	*p* Value	
	PTC	FTC
Age at diagnosis	<0.001	<0.001
Female vs. male	<0.001	0.044
Primary tumor size	<0.001	<0.001
Lymph node metastases	0.012	<0.001
Capsular invasion	<0.001	<0.001
Distant metastases	<0.001	<0.001
TNM stage		
I	-	-
II	0.014	0.630
III	0.998	0.995
IVa	<0.001	0.008
IVb	<0.001	<0.001
Neck recurrence	<0.001	0.002
New distant metastases	<0.001	0.174

## Data Availability

The data presented in this study are available on request from the corresponding author. The data are not publicly available due to the GDPR privacy policy.
